# Cognitive skill training improves memory, function, and use of cognitive strategies in cancer survivors

**DOI:** 10.1007/s00520-021-06453-w

**Published:** 2021-08-09

**Authors:** Monique M. Cherrier, Celestia S. Higano, Heidi J. Gray

**Affiliations:** 1 Department of Psychiatry and Behavioral Sciences, University of Washington, Box 356560, Seattle, WA 98195, USA; 2 Fred Hutchinson Cancer Consortium, Seattle, WA 98195, USA; 3 Department of Medicine, Division of Oncology, University of Washington, Seattle, WA 98195, USA; 4 Fred Hutchinson Cancer Research Center, Seattle, WA 98195, USA; 5 Department of Obstetrics and Gynecology, University of Washington, Seattle, WA 98195, USA

**Keywords:** Cancer, Oncology, Cognition, Cognitive training, Skills training, Group, Workshop, Controlled trial, Attention, Working memory, Active control, Education control

## Abstract

**Background:**

Cancer survivors commonly report symptoms of impaired cognition. This project examined effectiveness of a behavioral skills training intervention to improve cognition and reduce cognitive dysfunction symptoms in cancer survivors.

**Methods:**

Participants were randomly assigned to group-based workshops focused on learning new cognitive skills (skills treatment—TX) or an active control of education workshops (education control—EC) or a passive control of wait list (WL). Participants were evaluated pre- and post intervention with subjective mood and symptom questionnaires and objective neurocognitive tests.

**Results:**

One hundred twenty-eight participants (mean age 59 years), average 4.6 years (+/− 5.5 years) post cancer treatment with various cancer types (breast, bladder, prostate, colon, uterine), were enrolled. Analysis of all participants who attended workshop(s) revealed improvement in the TX group on all objective cognitive measures (attention, concentration, declarative, and working memory) save one test of selective attention, and improvement on a single measure (verbal memory) and decline (selective attention) in the EC group. TX group also improved on all symptom and mood measures, compare to EC group which improved on a single subscale of a symptom measure, but increased on an anxiety measure. TX group alone improved on a quantified measure of each participants’ unique, “top three,” self-described cognitive symptoms.

**Conclusion:**

Improvement from behavioral skills training was evident from objective cognitive tests, subjective symptom measures, and quantified, individual patient-specific symptoms. Behavioral skill training is an effective treatment for cognitive dysfunction in cancer survivors, and should be considered as a treatment option by health care providers.

## Introduction

It is estimated that there are 16.9 million cancer survivors in the United States, and approximately 17–75% report experiencing cognitive deficits, some which are severe enough to interfere with basic activities of daily living and work [1–3]. Common areas of cognitive impairment may include memory, attention, language, executive functions, and speed of information processing [4]. Despite numerous studies demonstrating significant cognitive impairments in a portion of survivors, research into effective treatments for cognitive difficulties is an emerging area of enquiry [5, 6].

This study examined the effectiveness of a behavioral skills training intervention to improve objectively measured cognitive performance as well as individual participant defined cognitive symptoms in cancer survivors. Several prior studies have shown the potential benefit of behavioral interventions focused on improving cognition [7–13] with indication that a focus on skills training is particularly beneficial [14] (see review by Von Ah & Crouch [5]). To improve upon limitations of prior intervention studies, the research design included provisions to reduce the potential for practice effects [15] from repeat cognitive testing and use of an education control group to address structural equivalency of the control condition such as possible treatment expectancy, staff contact, and social support confounds [16–18]. Both the education control (EC) and behavioral skills training (TX) were delivered in person, group, workshop style format. We hypothesized that behavioral cognitive skill training (TX) would result in improved objectively measured cognitive abilities and reduced cognitive symptoms compared to baseline, whereas EC and WL would not demonstrate a significant change from baseline.

## Materials and methods

### Subjects

Participants were adult cancer survivors recruited from the community through referral from health care providers or support organizations or via response to posted flyers. Inclusion criteria: (1.) Subjective concern about declines in cognitive functioning related to a diagnosis of cancer and/or cancer-related treatment. This was obtained during phone screening by requiring a yes response to the question: “do you have concerns about your memory or other thinking abilities related to your cancer or cancer treatment?” (2.) Age greater than 18 years and less than 90 years. (3.) Completion of active treatment for cancer (e.g., chemotherapy, radiation therapy, and surgery) 6 months or more in the past. Adjuvant therapies such as hormone receptor modulators, inhibitors, or other targeted therapies were allowed, as well as non-CNS radiation treatment(s). (4.) Able to read English and participate in informed consent process. Exclusion criteria were as follows: (1.) Ongoing treatment for cancer (e.g., chemotherapy, radiation, and surgery). (2.) Unstable medical problems. (3.) History, or current symptoms, of serious psychiatric disorder requiring antipsychotic medications or hospitalization. (4.) Current unhealthy alcohol use. (5.) History of or current neurological illness that significantly impacts cognition or prior significant brain injury. (6.) History of a central nervous system (CNS) tumor or CNS radiation. (7.) A score of 25 or more on the Patient Health Questionnaire (PHQ-9), a measure of depression in which a higher score indicates more depression symptoms [19]. (8.) A score of 26 or below on the Mini Mental Status Exam (MMSE), a screening measure of cognition in which a higher score is better functioning [20]. (9.) A score of 45 or more on the Wender Utah Rating Scale for attention deficit (WURS) in which a higher score indicates greater number and severity of symptoms [21].

### Procedures

The study design was a randomized, unblinded trial of a group-based cognitive training intervention. Participants underwent a phone screening followed by an in-person screening session (visit 1) including written informed consent, neurocognitive tests, and symptom questionnaires. Eligible participants were asked to return within 2 weeks for a second baseline assessment (visit 2) of repeat neurocognitive tests to help reduce practice effects. A subset of participants with gynecologic or breast cancer were eligible to participate in additional procedures of neuroimaging, results to be reported elsewhere.

**Figure 1.**
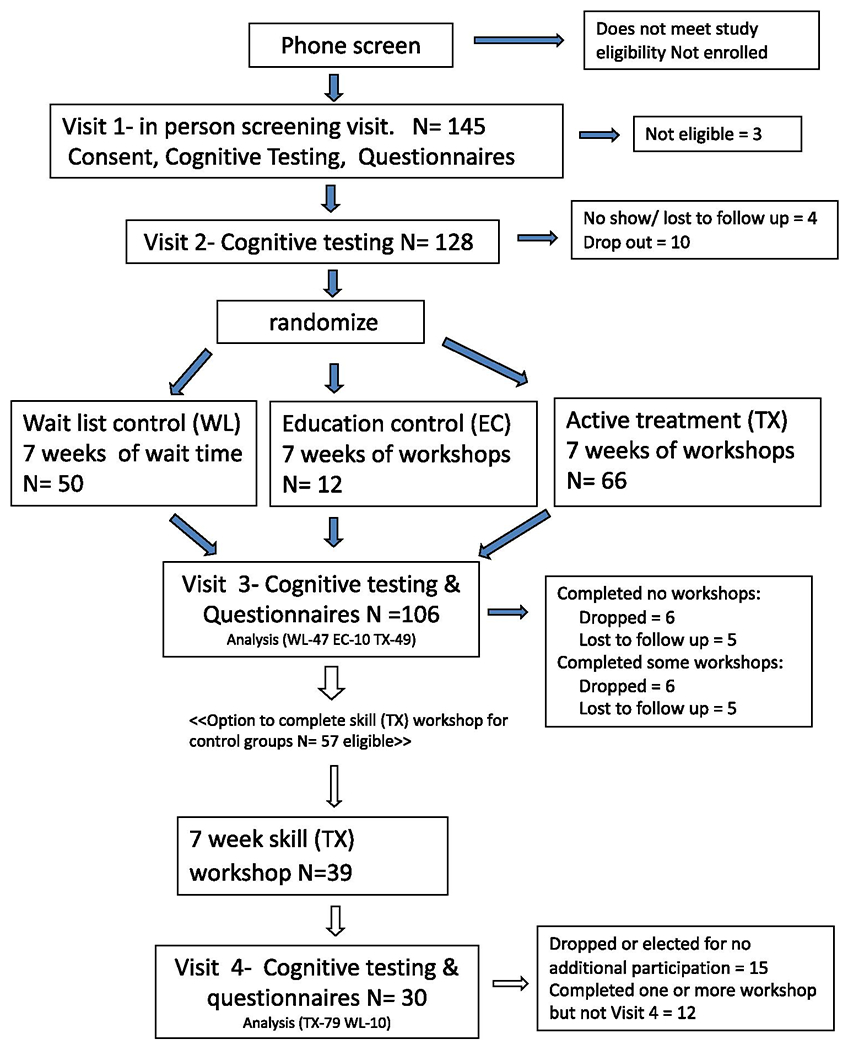
Consort flow chart

Following visit 2, participants were randomized according to one of three study conditions: active treatment (TX) composed of 7 weeks of behavioral cognitive skill training group workshops, or a comparable active control condition, education control (EC) composed of 7 weeks of education workshops on general cognitive science principals, or wait list (WL) control composed of a waiting period of 7 weeks without active contact from study staff. Education control condition was added after initial institutional review board (IRB) approval and start of enrollment, resulting in smaller group size compared to WL or TX due to lag in regulatory review and approval. After completing a study condition, participants underwent the same battery of objective cognitive tests and questionnaires at visit 3, and participants assigned to a control condition (EC and WL) were given the opportunity to participate in the active treatment (skills building) workshops. Participants who elected to continue in the study underwent objective cognitive tests and symptom questionnaires at visit 4 following the skills workshop, see Figure 1. Staff was not blinded to randomization condition, as it is not possible to conduct a specific behavioral intervention without knowledge of the condition assignment. Randomization order was created using an online number generator. To ensure objectivity during testing visits, workshop facilitators did not conduct post treatment assessment sessions.

### Baseline measures

A set of questionnaires to examine baseline characteristics and possible mediating influences on outcome were given at visit 1. These included the following: need for cognition (NFC), a measure of affinity toward cognitive challenging tasks [22], a shortened version of Dweck mindset quiz which assesses relative endorsement of “fixed” versus “growth” mindset [23], and an adapted readiness for treatment questionnaire (RTQ) which indicates how strongly a participant endorses statements reflective of a readiness to change [24], and Wechsler Test of Adult Reading (WTAR) for estimating general cognitive function [25].

### Symptom measures

Symptom-based measures were included at visits 1, 3, and 4. To assess uniquely individual cognitive symptoms, participants were asked to write down their “top three” most bothersome cognitive symptoms in their own words. For each cognitive problem, frequency (never to several times a day) and interference (not at all to very much) on a Likert scale were rated [26].

Additional symptom measures included Functional Assessment of Cancer Therapy-Cognition (FACT-Cog) [27], Patient Health Questionnaire (PHQ-9) [28], Beck Anxiety Inventory (BAI) [29], Fatigue (FACIT-Fatigue) [30], Attentional Function Index (AFI) [31], Meta-memory Questionnaire (MMQ) [32], and Patient’s Own Assessment of Functioning (PAOF) [33].

### Objective cognitive measures

The neurocognitive battery was given at visits 1, 2, 3, and 4 and used alternate test versions for all repeated tasks. Measures included Wechsler Adult Intelligence Scale-III (WAIS-III) subtests digit span, a measure of attention; digit symbol, a measure of sustained attention; letter-number sequencing, a measure of working memory [34]; Stroop task, a measure of selective attention [35]; modified three learning trial version of Rey Auditory Verbal Learning test (RAVLT-R), a measure of verbal memory [36]; and story recall, a measure of contextual verbal memory [37, 38]. Descriptions of tests and measures can be found in supplemental digital content #1 titled “ESM_#1.”

### Treatment fidelity, adherence, and participant satisfaction

To assess participant satisfaction with the workshops, participants were given a questionnaire at the final workshop [26]. To measure treatment fidelity and adherence, after each workshop session, participants rated instructor adherence to workshop curriculum, and a self-rating of content comprehension [26].

### Workshop procedures and content

Treatment (TX) and education control (EC) included seven workshop sessions each lasting 75 min and delivered over seven consecutive weeks. The focus of the treatment workshops centered on teaching new cognitive skills to address cognitive symptoms and concerns of cancer survivors. Content of the treatment (TX) workshops included use of memory aids, development of memory skills, working memory strategies, attention, mindful meditation, and overall brain health guided by neuroscience and behavioral research findings [39–42]. Content of education workshops (EC) included general concepts of human cognition, working memory, declarative memory, interaction of emotions and memory, attention and mindfulness, and impact of positive thinking. All workshops followed a standard outline starting with review of homework assignment, followed by a didactic portion in which new concepts were introduced, and ended with a homework assignment. For TX workshops, time was spent to practice new skills and for EC, time was spent in group discussion of didactic material. Workshops were conducted in person by trained facilitators with a limit of 12 participants. The wait list (WL) control condition involved no intervention.

### Statistical analysis

Data was entered into SPSS v.25 statistical software and double checked for accuracy [43]. Between-group comparisons (ANOVA, *T*-tests, chi-square) were conducted on demographic information, treatment fidelity, adherence, and other variables of interest that were non-repeating. To measure impact of treatment intervention, all participants who completed a workshop were included in a linear mixed model analysis with random effects of pre/post change over time (visits 2 and 4) and group (EC, TX). This primary comparison of workshop participants, ensured observations from both conditions, had comparable acquaintance to research staff, social exposure (other workshop participants), activity participation (e.g., workshop activities and homework), and potentially outcome expectations. Repeated measure mixed, linear mixed model with random effect analyses was used to measure pre/post change over time (visits 2–4) in all participants who completed treatment workshops (TX) and the education control (EC) group, interaction effects (treatment by visit), and between-group effects. All participants who completed the post intervention evaluation were included in the analysis regardless of number of workshop sessions completed. Covariates in the model for cognitive tests included age, education, post MMQ score, homework time, workshop sessions attended, and pre-baseline (visit 1) performance for each cognitive test. Symptom and mood measures included age as a covariate. Analyses with the same aforementioned parameters including TX, WL, and EC as grouping factor and change over time (visits 2–3) with Bonferroni correction for between-group comparisons were also conducted. “Results” section below includes significant findings, and non-significant findings are not reported, with the exception of validating assumptions (e.g., equivalency of group demographics following randomization).

## Results

One hundred forty-five participants passed the phone screening and were asked to come in for visit 1. After randomization, 50 participants were assigned to wait list (WL) control, 66 to active treatment workshops (TX), and twelve participants to education control (EC) workshops, see Figure 1. There were no significant differences between the treatment and control groups at baseline on any of the questionnaires, tests, or demographic variables. See Table 1 for demographic information.

### Neurocognitive tests

**Figure 2.**
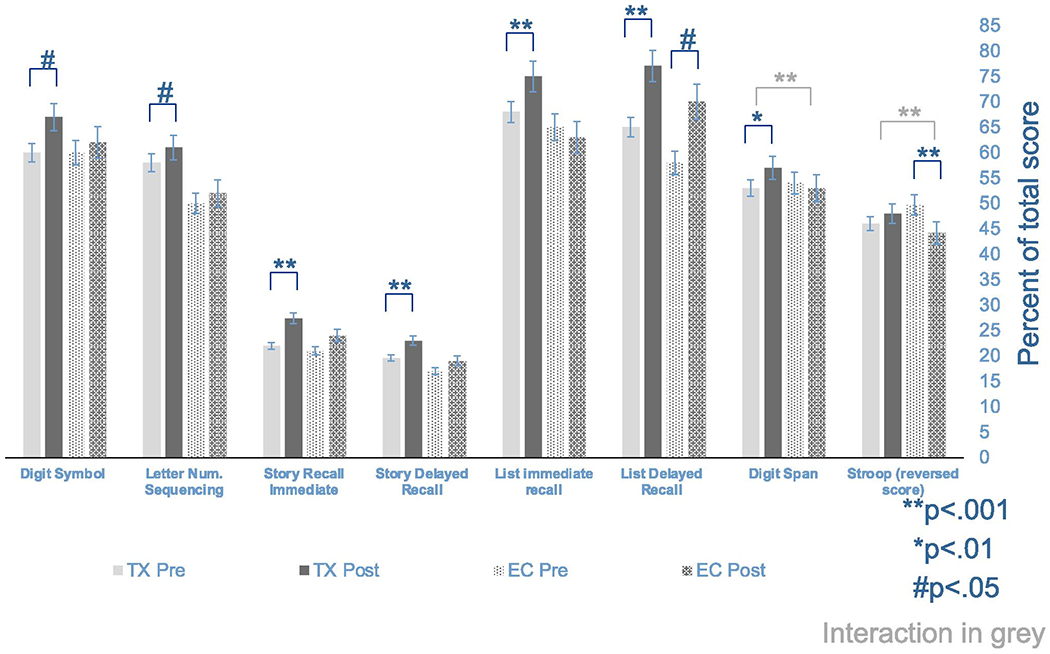
Participants who completed cognitive skills workshop (TX) compared to participants who completed education workshop (EC) on objective cognitive tests. Bars are mean, percent of total score possible for each measure with higher indicating better performance on all cognitive tasks (Stroop scores are reversed to allow for higher percent score to indicate better performance). Asterisks and pound sign indicate within-group significant change from baseline, and grey bars indicate significant interaction effects between groups. Error bars indicate standard error of mean (SEM).

Comparison of all TX workshop completers to EC at visit 4 revealed significant improvement from baseline on all cognitive tests for TX workshop completers (digit symbol, letter-number sequencing *p* < .05; story recall immediate and delayed, list recall immediate and delayed *p* < .001; digit span *p* < .01) except for Stroop, and improvement on delayed list recall (*p* < .05) but decline on Stroop (*p* < .01) for EC group. Interaction effects were observed for Stroop (*p* < .05), and for immediate list recall (*p* < .01) with decline in EC and improvement in TX workshop completers for both interactions, see Figure 2.

Comparison of all three groups (WL, EC, TX) at visit 3 revealed improvement in the TX group on story recall immediate and delay (*p* < .01), word list recall immediate and delay (*p* < .01), digit span (*p* < .01), Stroop, and letter-number sequencing (*p* < .05) and no significant change on digit symbol. WL improved on story recall immediate and delay and list delayed recall (*p* < .01) and EC declined on Stroop (*p* < .01). Interaction effects were observed on digit span (*p* < .01) with improvement in TX and steady and/or slight decline in EC and WL and interaction on Stroop (*p* < .01) with improvement in TX and WL and decline in EC.

### Questionnaires and symptom-based measures

**Figure 3.**
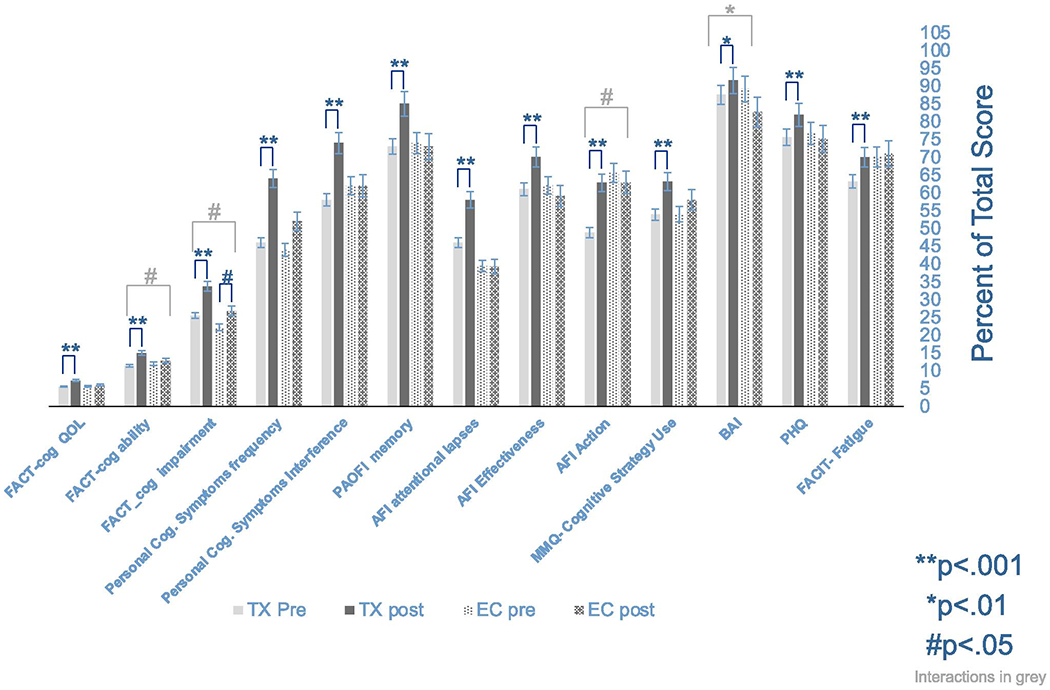
Participants who completed cognitive skills workshop (TX) compared to participants who completed education workshop (EC) on symptom and mood questionnaires. Bars are mean, percent of total score possible for each questionnaire with higher indicating improvement (i.e., reduced adverse severity or frequency rating) with scores reversed where needed for uniformity in presentation. Asterisks and pound sign indicate within-group significant change from baseline, and grey bars indicate significant interaction effects between groups. Error bars indicate standard error of mean (SEM). FACT, Functional Assessment of Cancer Therapy-Cognition (FACT-Cog) — subscale scores, perceived cognitive impairments — higher score indicates fewer adverse symptoms, QOL — higher score equals higher cognitive quality of life, perceived cognitive abilities — higher score indicates a higher perception of abilities, personal cognitive symptoms — frequency average score of frequency rating across top three symptoms higher indicates less frequent occurrence, interference — average of severity rating across top three symptoms, higher score indicates less severity, PAOFI, Patients Own Assessment of Functioning Memory subscale — higher is less memory disturbance and difficulties, Meta-memory Questionnaire (MMQ) — strategy use subscale higher score indicates more use of memory strategies, Attention Function Index (AFI) effective action (EA) subscale, interpersonal effectiveness subscale (IE) attentional lapses subscale (AL) higher score is better functioning in these areas, Beck Anxiety Inventory (BAI) — total score higher score indicates more severe anxiety symptoms; (PHQ-9) total score, higher score indicates less severe depression symptoms; Functional Assessment of Chronic Illness Therapy-Fatigue (FACIT-Fatigue) — total score, higher score equals less fatigue.

Similar to results for objective tests, the primary analysis of TX workshop completers and EC revealed significant improvement for TX group on all questionnaire and symptom measures (*p* < .001), and significant improvement for BAI at *p* < .01 significance level. EC improved on FACT-Cog perceived cog. impairment (*p* < .05) and increased on BAI (i.e., more anxious) (*p* < .05). Interaction effects were observed with both groups (TX, EC) improving, but a greater degree or amount of improvement observed in the TX workshop completers was observed on the FACT-Cog ability and impairment subscales (*p* < .05). Significant interaction effects with improvement in the TX workshop completers but decline in the EC group were observed for AFI-effective action and BAI, see Figure 3.

Comparison of all three groups (TX, EC, WL) at visit 3 revealed significant improvement for TX group on all measures (*p* .01) except BAI. EC improved on FACT-Cog perceived cognitive impairment (*p* < .05), and WL improved on AFI-effective action subscale (*p* < .05). Interaction effects were observed for all measures (*p* < .01) with the exception of BAI, in the direction of improvement in the TX group, and less improvement, steady performance, and/or decline in EC and WL groups. Direction of improvement, maintenance, and decline of each measure and associated significance for change over time for all groups and measures can be found in online resource #2 titled “ESM_#2.”

### Adherence and treatment fidelity

Following each workshop session, participants completed a questionnaire rating the instructor/workshop leader on instruction and participant comprehension on a Likert scale (zero to 10). Between-group comparisons (TX, EC) revealed no differences between the groups on these items with the exception of participant rating of the workshop leader’s teaching in which the rating for the treatment group was higher than the education control group (*p* < .01). There were no significant differences between the treatment (TX) and control (EC) groups on measures of adherence including number of workshops attended, and mean amount of time spent on homework. See data details in online resource #2 titled “ESM_#2.” Post workshop satisfaction questionnaire responses are provided in online resource #3 titled “ESM_#3.”

## Discussion

This study examined the effectiveness of a workshop format, skills training intervention to improve cognition and reduce cognitive dysfunction symptoms in cancer survivors. Cancer survivors who completed treatment workshops (TX) improved on both objective cognitive tests and symptom-based questionnaires. Improvements on objective cognitive tests were observed on multiple measures of verbal memory (word list and story recall) including both immediate and delayed recall, attention (digit span), sustained attention (digit symbol), and working memory (letter-number sequencing), controlling for age, education, strategy use (MMQ), time spent on homework, workshop attendance, and baseline performance. In contrast, the EC group improved on only one measure of verbal memory (word list delayed), but not immediate word list recall, and declined on a measure of selective attention (Stroop). WL group improved on a measure of verbal memory and attention

In addition to improvement on objective measures of cognitive function, a major indicator, of meaningful improvement on cognition for participants of the skill-based workshops, was the significant improvement from baseline on their self-selected cognitive symptoms. Participants were asked to select and describe in their own words, their “top three” most bothersome cognitive symptoms, and rated the severity and frequency of each symptom. Post workshop (TX, EC) or wait list (time), participants again rated the severity and frequency of their self-selected symptoms. Only the treatment group (TX) decreased in frequency and severity of their self-selected and self-described cognitive dysfunction symptoms. Neither EC nor WL groups changed on this measure. However, for WL participants that elected to participate in the treatment workshop, a significant decrease in severity and frequency of their self-selected and described symptoms was observed post skill-based workshop (TX) completion. This suggests that a skill-based workshop can provide relief of participants’ most troublesome and uniquely individual symptoms.

Observation of improvement on individual symptoms is important both to cancer survivors and third party stakeholders who may believe that patients must demonstrate a specified level of “impairment” to be considered eligible for treatment or that a specified level of improvement (e.g., two standard deviations) must be demonstrated for a treatment to be considered effective or viable. This measure also provides additional specificity and precision in comparison to general, or composite symptom inventories or global clinical impression of change measures, which can be influenced by a number of un-related factors, as well as the potential to be insensitive to patient-specific symptoms.

Similar workshop style interventions in cancer survivors have also reported improvements on objective tests of verbal memory [7], attention [44], and general, composite scores from cognitive batteries [12]. However, to our knowledge, these prior studies have not included comparison to an active control condition (i.e., education control) or measures of treatment adherence and fidelity, or efforts to minimize practice effects such as a double baseline.

The inclusion of an active control group (education control—EC) in which all of the non-specific treatment factors are present, with the exception of the active treatment component, provides a strong study design and is a recommended practice in behavioral interventions to help control nuisance factors. In this study, the education control (EC) group had the same group workshop treatment environment, followed the same general session outline, and included homework assignments, as did the active treatment (TX) condition. However, EC content did not include cognitive compensatory skill instruction. Only the TX group demonstrated an increase in use of memory and cognitive strategies as measured by the MMQ questionnaire (cognitive strategy subscale) compared to the EC group, which suggests that improvement in objective cognitive tests and symptom measures is associated with learning, and increased use of new cognitive skills and techniques rather than non-specific treatment factors.

Improvements in mood, anxiety, and fatigue symptom measures were observed in the TX group but not anticipated, as the cognitive skill workshops did not include skills or information directly addressing mood or fatigue symptoms. An indirect connection is possible, as information on mindful meditation and maintaining good brain health which makes some connections between sleep, stress, physical activity, and cognition was included in the cognitive skills (TX) workshop content. However, the education workshop content contained modules about the immune system and the mind which also makes connections between stress and cognition, but improved mood or fatigue was not observed, rather an increase in anxiety. Participants in the cognitive skills workshops may have made some self-improvement changes in these areas that could be reflected in part by the mood and fatigue measures. It is also possible that improvements in cognition from the skill-based treatment intervention reduced the frequency of bothersome cognitive symptoms which indirectly impacted mood and fatigue, as improved mood and fatigue were only observed in the TX group. Clinically significant elevations in depression, anxiety, and fatigue often co-occur with impaired cognitive symptoms, and have been observed prior to, during, and following cancer treatment as well as in non-cancer samples [45–48].

Results are limited by small sample size in the active control (education control—EC) group. While the study can be strengthened by a larger sample size in the active control condition, it is not unusual to have a smaller sample size in the control condition for treatment studies, particularly behavioral treatment studies where there is more effort and cost involved in delivering the treatment and where a crossover design is not feasible. In addition, the small size of the EC group is further mitigated by the size of the WL control group.

Participants in this study were enrolled based on self-endorsement of cognitive dysfunction. An examination of baseline cognitive scores reveals performance ranging from mild weakness to normal and above average performance. It is possible that improvement in all treatment groups might have been more robust by restricting enrollment to participants who met some objective criteria of “impairment” at baseline. However, this selection approach was not utilized in this sample of cancer survivors for several reasons including the following: (1) It was anticipated that enrolled participants were not likely to perform above average on *all* objective measures, and therefore, there would be room for improvement in select abilities. (2) Given that many cancer survivors are older and the risk of dementia increases with age, studies of cancer survivors that select for cognitive deficits may be over-inclusive of patients with dementia or incipient dementia which is a progressive disorder. (3) Participants rated the frequency and severity of their own unique “top three” cognitive symptoms at baseline and again after the treatment workshop. Thus, the study obtained a uniquely individual symptom measure against which change over time could be calibrated independent of a uniform baseline functioning requirement. (4) Pre-baseline performance scores (visit 1) were included as a covariate in analysis models, providing another control factor in the analysis models.

This study did not include a formal analysis with regard to missing data biases. However, there were no significant differences between skills workshop (TX) and education control (EC) groups for the percent of workshops attended. Treatment fidelity factors including participant rating of instructor adherence to workshop outline and participant self-rating of workshop learning were comparable between TX and EC groups, except for one question in which EC group rated leader instruction during workshops as slightly lower (mean 8.5 out of ten) than TX group (mean 9.4 out of ten), which may have impacted outcomes. Although it may also be related to the degree to which leading a discussion around educational content compared to skill instruction lends itself toward different ratings of instructor effectiveness.

## Clinical implications

These results suggest that workshop style cognitive skills training is an effective treatment for cancer survivors who are struggling with symptoms of cognitive dysfunction. The treatment was effective for cancer survivors with all types of cancers and was effective in improving symptoms unique to each participant. Our results are consistent with previous findings of improved cognition from behavioral, in person, group, workshop style interventions and expand these findings with the addition of strong study design elements that provide better control of confounds, and increased confidence of results as well as assessment of treatment fidelity and participant adherence. This approach should be incorporated into survivorship programs and further studied. Health care professionals should consider referral for cognitive skill training for cancer survivors who report cognitive dysfunction symptoms, including patients who report both mood, anxiety, and cognitive dysfunction as the treatment resulted in significantly improved mood and improved fatigue as well as cognition.

## Supplementary Material

1745037_Sup~file1

1745037_Sup~file2

1745037_Sup~file3

## Figures and Tables

**Table 1.** Demographic information (mean, S.D.)

	Treatment (TX)	Wait list control (WL)	Education control (EC)	Total	F, *χ*^2^
*N*	66	50	12	128	
Age (years)	59 (9)	59 (12)	61 (10)	59 (11)	0.346, NS
Education	16.9 (2.5)	16.6 (2.5)	17.0 (3.5)	16.8 (2.5)	0.402, NS
MCQ	13.1 (10.4)	13.6 (10.0)	13.2 (6.1)	13.3 (9.9)	0.025, NS
Sex	F = 56; M = 10	F = 40; M = 10	F = 10; M = 2	F = 106; M = 22	0.790, NS
Years since treatment	4.5 (5.3)	4.5 (5.7)	3.7 (3.3)	4.4 (5.3)	0.117, NS
*Types of treatment*					
Chemotherapy	44%	36%	9%	89%	1.39, NS
Radiation	30%	20%	3.9%	54%	2.23, NS
Surgery	41%	33%	9.7%	84%	4.78, NS

*NS*, non-significant, *F*-value from analysis of variance or *χ*^2^, chi-square value for between-group (TX, WL, EC) comparisons.

## Data Availability

Data available on request from the authors. See information at supporting information sites noted above.
