# Supplementary material for: Cognitive skill training improves memory, function, and use of cognitive strategies in cancer survivors
Source: Support Care Cancer. Author manuscript; Available in PMC 2023 Jan 1. (PMC8639759; doi:10.1007/s00520-021-06453-w)
Supplement: 1745037_Sup~file1 [file NIHMS1745037-supplement-1745037_Sup_file1.pdf]

Screening or Baseline only measures:

MMSE- Mini-mental Status Examination (MMSE). A brief mental status examination that assesses language, attention, orientation, calculation and comprehension.[1] Higher score indicates better performance. The measure has been used for screening of older adults suspected of neurological disorders including dementia.[2]

WURS- Wender Utah Rating Scale (WURS) a measure of common attention deficit hyperactivity (ADHD) symptoms designed to be given to adults.[3, 4] Symptoms are rated on a likert scale and responses are scored according to a norm based rating system. Higher score indicates greater number and severity of ADHD symptoms.

WTAR: Wechsler Test of Adult Reading (WTAR) a measure that provides a general estimate of overall verbal and general cognitive abilities, and is resistant to changes from age and disease. Higher score indicates a higher general level of functioning.[5]

NFC- The Need for Cognition (NFC) questionnaire that assesses to what degree someone considers cognitive challenging situations as aversive. A higher score indicates a stronger rating of aversive for cognitively challenging situations.[6]

Dweck Mindset quiz: A shortened (seven item) version of the mindset quiz developed by Carol Dweck was used to determine growth versus fixed mindset.[7] Participants rate items relating to cognitive mindset (fixed or growth) on a likert scale (strongly agree to strongly disagree). A

higher score indicates a stronger belief of growth versus fixed mindset toward cognitive abilities such as memory, attention, learning and intelligence.

RTQ- Readiness for treatment questionnaire (RTQ), was adapted from measures related to psychotherapy treatment and refined to apply toward treatment related to cognitive symptoms, to characterize the particular stage of change or readiness for change and treatment.[8] Participants rate statements about need for change and cognitive symptom severity on a likert scale (disagree to agree). Items are scored with a final assignment of one of three categories (pre-contemplation, contemplation or action) according to highest score.

Other questionnaires- and Treatment Fidelity and Adherence Measures. Copies of these measures can be found at https://osf.io/y83bd/
Workshop satisfaction: 'post_treatment_questionnaire' A questionnaire on satisfaction with the workshops, which was given at the final workshop session.

Treatment Fidelity: "participant_eval_of_instructor'. Participants were given a questionnaire at the end of each workshop session, that asked participants to rate on a likert scale the degree to which the workshop leader, covered the content for that day's workshop. Question also asked how well the participant comprehended the content.

Adherence: 'instructor_rating_of_participant_effort'. Workshop instructors rated participants on a likert scale according to how well they reported using and applying the workshop material, as well as their impression of participation and effort in the workshops and for homework.

Repeat measures:

Symptom Measures:

FACT-Cog. Functional Assessment of Cancer Therapy-Cognition (FACT-Cog) [9]. The FACT-Cog measures frequency and interference of cognitive symptoms. It has three subscales:

symptoms of perceived cognitive impairments with higher indicating fewer symptoms, perceived cognitive abilities in which a higher score indicates a rating of better cognitive abilities, and overall quality of life with a higher score indicating better quality of life as it relates to cognition.

Top Three Cog. Symptoms: Each participant is asked to write a description of their top three, most bothersome cognitive symptoms. For each cognitive problem they indicated on a likert scale, frequency (never to several times a day) and interference (not at all to very much). Higher scores indicate more severe and more frequent occurrence of the cognitive symptoms. A copy of the questionnaire and samples can be fount at: https://osf.io/y83bd/

PHQ-9: The Patient Health Questionnaire (PHQ-9) a measure of depression symptoms, for which a higher score indicates more symptoms of depression [(10)].

BAI: Beck Anxiety Inventory (BAI), an anxiety symptom measure in which a higher score indicates endorsement of more and/or more severe anxiety symptoms. [(11)]

FACIT- fatigue: Functional Assessment of Chronic Illness Therapy – Fatigue (FACIT- Fatigue), measures fatigue symptoms, with higher scores indicating a better quality of life and fewer fatigue symptoms [(12)]

AFI: Attentional Function Index (AFI) a measure of attention and distraction that was developed for use with cancer patients, with higher scores indicating better attention.[(13)] There are three AFI

subscales: interpersonal effectiveness (IE) relates to control from distractions, effective action (AE) relates to multi-tasking, and attentional lapses (AL) relates to instances of lost attention.

MMQ: Multiphasic Memory Questionnaire (MMQ) a comprehensive measure of memory that includes a subscale of use of memory strategies. Only the strategy subscale of this measure was included to determine the degree to which participants utilized memory strategies. Higher scores indicating more frequent use of memory strategies and aids.[(14)].

SF-36: SF-36 health survey, a general quality of life measure that contains subscales of general health, limitations of activities, physical health, emotional health, social activities, pain, energy and emotions, and social activities along with a total score with higher scores indicating better functioning.[(15)]

PAOF: The patient's assessment of own functioning (PAOF) a measure of perceived disability relating to cognition, with subscales of memory, language-communication, hands, sensory-perceptual, and higher-level cognitive functions with higher score indicating more problems and lower score fewer problems. The memory subscale was used. [(16)]

Objective Cognitive Measures:

The neurocognitive battery was comprised of standard objective measures of attention, memory, and executive function, and use of alternate test versions was used. The test battery was given at all study visits (1, 2, 3 and for some participants visit 4).

WAIS-3- Subscales: Wechsler Adult Intelligence Scale -III(WAIS-III) [17].

Digit Span: Digit span is a task of attention and working memory and involves hearing a series of digits and recalling them in the same order (forward) or in the reverse order (backward). A score is given for both forward and backward and a total score is generated with a higher score indicating better performance.

Letter number sequencing: Letter- number sequencing a task of attention and working memory, participants listen to a random sequence of numbers and letters and must reply with the same information given in numerical and alphabetical order.

Digit symbol: Digit symbol is a task of psychomotor coordination, visual tracking, and working memory and involves rapid completion of a series of symbols according to a visible key, with higher scores indicating better performance.

Stroop: Stroop test, a task of executive function, involves reading text, naming color blocks and the interference trial in which the pre-potent response of reading must be inhibited to name ink color. Time to complete is recorded so that a lower score is better performance [18].

RAVLT-R: The Rey Auditory Verbal Learning test revised (RAVLT-R), is a task of verbal memory in which participants hear a word list and must recall it after several presentations and a short delay [19]. A modified version of this test was used in which only three learning trials were administered, followed by the interference trial, immediate recall and delayed recall. Total recall across trials as well as the delay are recorded with a higher score indicating better verbal memory.

Story Recall: To measure contextual verbal recall (i.e., story recall) participants listened to a short story, and recalled the story immediately and after a 30 minute delay. The amount of information recalled from the story was scored using a standardized scoring method, with a higher score indicating better recall. For visit 1, participants were given part a of Wechsler Memory Scale-Revised Logical memory[(20)] and subsequent study visits included alternate paragraphs developed by Sullivan et al. [(21)]

1. Folstein MF, Folstein SE, McHugh PR. "Mini-mental state";. A practical method for grading the cognitive state of patients for the clinician. J Psychiatr Res. 1975;12(3):189-98.
2. Clarke M, Jagger C, Anderson J, Battcock T, Kelly F, Stern MC. The prevalence of dementia in a total population: a comparison of two screening instruments. Age Ageing. 1991;20(6):396-403.
3. Ward MF, Wender PH, Reimherr FW. The Wender Utah Rating Scale: an aid in the retrospective diagnosis of childhood attention deficit hyperactivity disorder. Am J Psychiatry. 1993;150(6):885-90.
4. Jaffe C, Bush KR, Straits-Troster K, Meredith C, Romwall L, Rosenbaum G, et al. A comparison of methamphetamine-dependent inpatients with and without childhood attention deficit hyperactivity disorder symptomatology. Journal of Addictive Diseases. 2005;24(3):133-52.
5. Wechsler. Wechsler Test of Adult Reading (Manual). San Antonio, TX: The Psychological Corporation; 2001.
6. Cacioppo JT, Petty RE, Kao CF. The Efficient Assessment of Need for Cognition. Journal of Personality Assessment. 1984;48(3):306-7.
7. Dweck C. Self-theories: Their role in motivation, personality, and development. Philadelphia: Psychology Press; 1999.
8. Mcconnaughy EA, Prochaska JO, Velicer WF. Stages of Change in Psychotherapy - Measurement and Sample Profiles. Psychother-Theor Res. 1983;20(3):368-75.
9. Jacobs SR, Jacobsen PB, Booth-Jones M, Wagner LI, Anasetti C. Evaluation of the Functional Assessment of Cancer Therapy Cognitive Scale with Hematopoetic Stem Cell Transplant Patients. J Pain Symptom Manage. 2007;33(1):13-23.
10. Wittkampf KA, Naeije L, Schene AH, Huyser J, van Weert HC. Diagnostic accuracy of the mood module of the Patient Health Questionnaire: a systematic review. Gen Hosp Psychiatry. 2007;29(5):388-95.
11. Stanley MA, Beck JG, Zebb BJ. Psychometric properties of four anxiety measures in older adults. Behav Res Ther. 1996;34(10):827-38.
12. Cella DF. Manual of the Functional Assessment of Chronic Illness Therapy (FACIT Scales) - Version 4. Evanston, IL: Center on Outcomes Research and Education (CORE); 1997.
13. Cimprich B, Visovatti M, Ronis DL. The Attentional Function Index--a self-report cognitive measure. Psychooncology. 2011;20(2):194-202.

14. Troyer AK, Rich JB. Psychometric properties of a new metamemory questionnaire for older adults. The journals of gerontology Series B, Psychological sciences and social sciences. 2002;57(1):P19-27.
15. McHorney CA, Ware JE, Jr., Raczek AE. The MOS 36-Item Short-Form Health Survey (SF-36): II. Psychometric and clinical tests of validity in measuring physical and mental health constructs. Med Care. 1993;31(3):247-63.
16. Chelune GJH, Robert K. Lehman, Ralph A. W. Neuropsychological and Personality Correlates of Patients' Complaints of Disability. Advances in Clinical Neuropsychology; 1986. p. 95-126.
17. Wechsler D. WAIS-III administration and scoring manual. San Antonio, TX: The Psychological Corporation; 1997.
18. Delis DC, Kaplan E, Kramer J. Delis-Kaplan Executive Function System. San Antonio, TX: Psychological Corporation; 2001.
19. Schmidt M. Rey Auditory Verbal Learning Test: A Handbook. Los Angeles, CA: Western Psychological Services; 1996.
20. Wechsler D. Wechsler Memory Scale -Revised. San Antonio, TX: The Psychological Corporation; 1987.
21. Sullivan K. Alternate forms of prose passages for the assessment of auditory-verbal memory. Arch Clin Neuropsychol. 2005;20(6):745-53.
