# Supplementary material for: Cognitive skill training improves memory, function, and use of cognitive strategies in cancer survivors
Source: Support Care Cancer. Author manuscript; Available in PMC 2023 Jan 1. (PMC8639759; doi:10.1007/s00520-021-06453-w)
Supplement: 1745037_Sup~file2 [file NIHMS1745037-supplement-1745037_Sup_file2.pdf]

**Online Resource #1.** Cherrier, Gray, Higano (2021) JSCC.

**Table . Demographics, Questionnaires, and Neurocognitive Results: Means and Standard Deviation**

| **Demographics** | **Treatment (Tx)** | | | **Wait List Control (WL)** | | | | **Education Control (EC)** | | | **Total** |
|---|---|---|---|---|---|---|---|---|---|---|---|
| *N* | 66 | | | 50 | | | | 12 | | | 128 |
| Age | 59.4 (10.2) | | | 58.5 (12.5) | | | | 61.3 (10.4) | | | 59.2 (11.1) |
| Education | 16.9 (2.5) | | | 16.8 (2.5) | | | | 17.0 (3.5) | | | 16.8 (2.5) |
| MCQ | 13.4 (SD = 10.3) | | | 14.1 (SD = 10.2) | | | | 13.2 (6.1) | | | 13.7 (9.9) |
| Sex | F = 56; M = 10 | | | F = 40; M = 10 | | | | F = 10; M = 2 | | | F = 106; M = 22 |
| Years Since Treatment | 4.6 (5.3) | | | 4.7 (6.1) | | | | 3.7 (3.3) | | | 4.6 (5.5) |
| *Types of Treatment* | | | | | | | | | | | |
| Chemotherapy | 44% | | | 34% | | | | 8% | | | 86% |
| Radiation | 31% | | | 19% | | | | 4% | | | 54% |
| Surgery | 43% | | | 31% | | | | 8% | | | 82% |
| **Measures** | V1 | V2 | V3 | V1 | V2 | V3 | V4 | V1 | V2 | V3 | |
| *Objective Cognitive Tests* | | | | | | | | | | | |
| Digit Span (total score) | 18.2 (4.0) | 19.8 (3.7) | **21.4 (4.2)*** | 17.2 (3.7) | 17.7 (3.8) | 17.5 (4.3) | 18.9 (4.0)* | 16.8 (4.6) | 17.0 (4.9) | 17.5 (3.6) | -- |
| Digit Symbol (score) | 71.6 (15.7) | 72.5 (14.8) | 74.4 (13.8)* | 67.6 (17.3) | 68.0 (15.6) | 65.7 (15.6) | 76.5 (14.1)* | 70.6 (10.5) | 70.6 (16.1) | 70.2 (14.3) | -- |
| Stroop Intereference (seconds to complete) | 57.2 (13.3) | 52.1 (13.1) | **51.0 (11.3)** | 60.6 (14.3) | 56.5 (11.6) | 55.9 (13.7) | 53.2 (12.5) | 63.8 (16.0) | 52.3 (12.5) | 60.0 (10.4) | -- |
| Stroop Interference Plus (seconds to complete) | 64.2 (17.3) | 67.9 (15.6) | 63.7 (14.5) | 71.2 (19.7) | 77.3 (24.5) | 73.0 (23.4) | 73.7 (16.5) | 82.2 (35.6) | 78.8 (16.1) | **84.5 (36.9)*** | -- |
| Letter Number Sequencing (score) | 11.4 (2.8) | 12.8 (2.7) | **13.6 (3.2)*** | 11.4 (3.4) | 11.6 (3.0) | 11.4 (2.7) | 12.2 (2.8)* | 10.8 (3.3) | 10.6 (3.3) | 10.9 (1.9) | -- |
| List Learning Immediate (total across trials) | 31.9 (6.8) | 32.7 (5.5) | **35.5 (6.5)*** | 29.0 (6.3) | 27.7 (7.0) | 29.5 (7.2) | 33.4 (6.4)* | 28.0 (8.1) | 27.6 (8.1) | 27.2 (8.6) | -- |
| List Learning Delay (total) | 10.3 93.2) | 10.6 (2.8) | **12.4 (2.3)*** | 9.1 (3.4) | 8.6 (3.5) | **9.9 (3.3)** | 11.0 (3.8)* | 8.2 (4.1) | 7.6 (4.9) | 9.1 (4.1)* | -- |
| Story Recall Immediate (score) | 11.8 (3.5) | 11.6 (3.3) | **14.7 (3.5)*** | 11.5 (3.4) | 10.5 (3.4) | **14.3 (4.3)** | 12.6 (3.6)* | 11.3 (3.4) | 10.8 (4.1) | 12.8 (4.1) | -- |
| Story Recall Delay (score) | 10.3 (3.5) | 10.1 (3.3) | **12.6 (3.6)*** | 9.3 (3.4) | 8.7 (3.2) | **11.7 (3.9)** | 11.2 (3.5)* | 10.2 (4.3) | 9.1 (4.3) | 11.0 (4.2) | -- |
| *Questionnaires* | | | | | | | | | | | |
| Fact: Perceived Cognitive Impairment | 36.8 (16.5) | - | **49.8 (13.4)*** | 38.8 (18.1) | - | 38.7 (17.7) | 49.5 (13.3)* | 33.9 (15.8) | - | **40.7 (21.1)*** | -- |
| Fact: Cognitive Quality of Life | 8.2 (4.6) | - | **11.3 (3.7)*** | 8.6 (4.6) | - | 8.8 (4.6) | 11.1 (4.1)* | 8.7 (4.3) | - | 9.6 (4.4) | -- |
| Fact: Perceived Cognitive Abilities | 16.7 (8.2) | - | **21.6 (6.1)*** | 16.8 (6.9) | - | 16.4 (6.7) | 22.0 (5.6)* | 18.3 (7.6) | - | 21.0 (7.1) | -- |
| Personal Cognitive Symptoms -Frequency | 2.8 (0.7) | - | **1.8 (0.7)*** | 2.6 (0.9) | - | 2.6 (0.8) | 1.8 (0.6)* | 2.8 (0.8) | - | 2.4 (1.0) | -- |
| Personal Cognitive Symptoms- Interference | 2.3 (0.9) | - | **1.3 (0.7)*** | 2.2 (1.1) | - | 1.9 (1.0) | 1.3 (0.8)* | 1.9 (0.9) | - | 1.8 (1.2) | -- |
| PAOFI - Memory (%) | 0.30 (0.19) | - | **0.15 (0.14)*** | 0.30 (0.20) | - | 0.28 (0.23) | 0.15 (0.14)* | 0.26 (0.23) | - | 0.28 (0.20) | -- |
| Meta-memory Questionnaire - Strategy Use | 41.7 (9.3) | - | **48.0 (7.8)*** | 40.3 (11.5) | - | 41.5 (11.3) | 47.4 (8.9)* | 41.0 (9.1) | - | 43.1 (12.4) | -- |
| Attention Function Index - EA | 46.4 (17.5) | - | **64.9 (15.3)*** | 50.4 (20.2) | - | **56.3 (20.0)** | 64.9 (15.3)* | 66.7 (17.3) | - | 58.0 (22.3) | -- |
| Attention Function Index- IE | 60.8 (21.5) | - | **71.4 (18.0)*** | 61.4 (19.6) | - | 62.9 (18.1) | 71.4 (18.0)* | 63.1 (24.1) | - | 59.4 (16.2) | -- |
| Attention Function Index- AL | 43.7 (21.0) | - | **58.1 (20.6)*** | 48.1 (24.8) | - | 46.4 (24.0) | 58.1 (20.6)* | 39.9 (19.2) | - | 39.8 (18.9) | -- |
| Beck Anxiety Inventory (BAI) | 6.7 (5.7) | - | 4.7 (3.5)* | 7.8 (7.6) | - | 7.2 (8.9) | 4.7 (3.6)* | 6.6 (6.2) | - | 10.0 (13.8)* | -- |
| Patient Health Questionnaire (PHQ-9) | 6.7 (4.5) | - | **4.4 (3.4)*** | 6.7 (5.1) | - | 6.9 (6.0) | 4.1 (3.3)* | 6.2 (4.8) | - | 6.6 (6.8) | -- |
| FACIT - Fatigue | 33.1 (11.4) | - | **38.5 (9.0)*** | 32.6 (10.1) | - | 33.6 (10.3) | 36.6 (9.3)* | 37.3 (8.7) | - | 37.1 (11.5) | -- |
| *Attendance, Treatment Fidelity* | | | | | | | | | | | |
| *Workshop Sessions Attended* | 5.6 (1.9) | | | | | | | 5.2 (2.4) | | | |
| Homework minutes per session | 18.5 (15.4) | | | | | | | 8.5 (12.3) | | | |
| participant self-rating of learning | 8.8 (0.6) | | | | | | | 7.8 (2.2) | | | |
| participant rating of leader instruction | **9.4 (0.5)** | | | | | | | **8.5 (2.6)** | | | |
| instructor adherence to workshop outline | 9.1(0.6) | | | | | | | 7.6 (2.0) | | | |

**BOLD text** significant change from baseline for three group (WL, EC, TX) analysis at visit 3. Attendance & treatment fidelity items are between group comparisons.

*significant change from baseline for two group analysis (TX,EC) at visit 4. TX group included all participants who completed a treatment workshop. Results for the combined TX group are indicated in visit 4 column for WL and visit 3 column for TX. refer to figure 1 for study flow details.
