# Supplementary material for: Cognitive skill training improves memory, function, and use of cognitive strategies in cancer survivors
Source: Support Care Cancer. Author manuscript; Available in PMC 2023 Jan 1. (PMC8639759; doi:10.1007/s00520-021-06453-w)
Supplement: 1745037_Sup~file3 [file NIHMS1745037-supplement-1745037_Sup_file3.pdf]

# Post Workshop Satisfaction Survey

Supplemental Electronic_ Information #3: Cherrier, Higano, Gray- JSCC, 2021

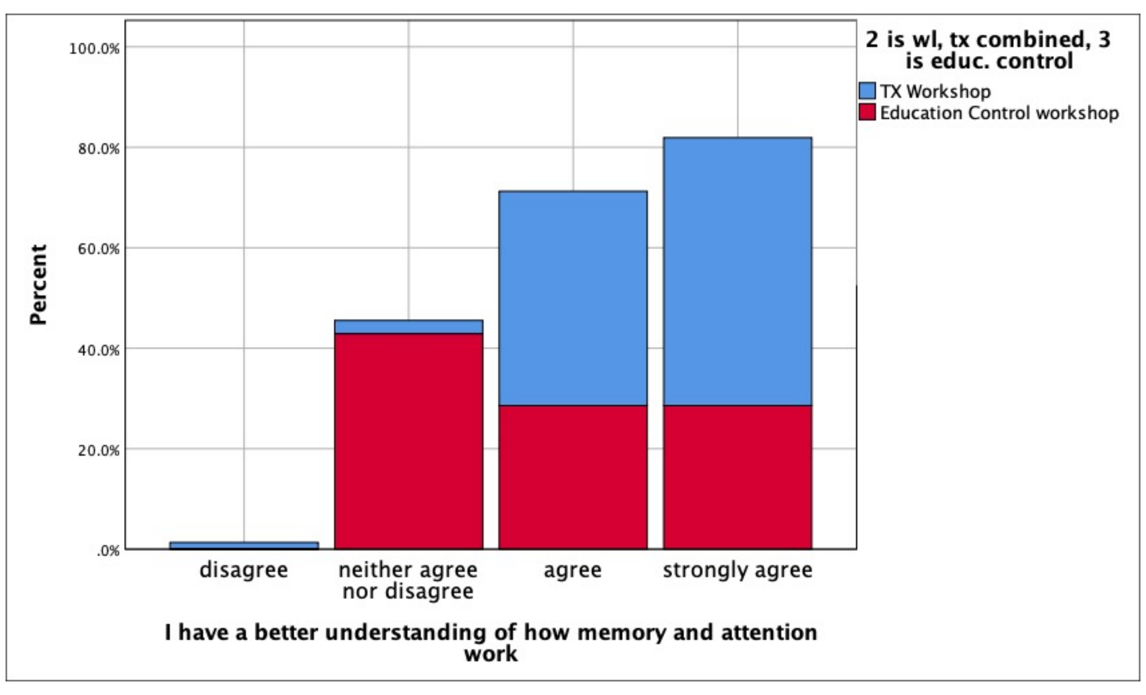


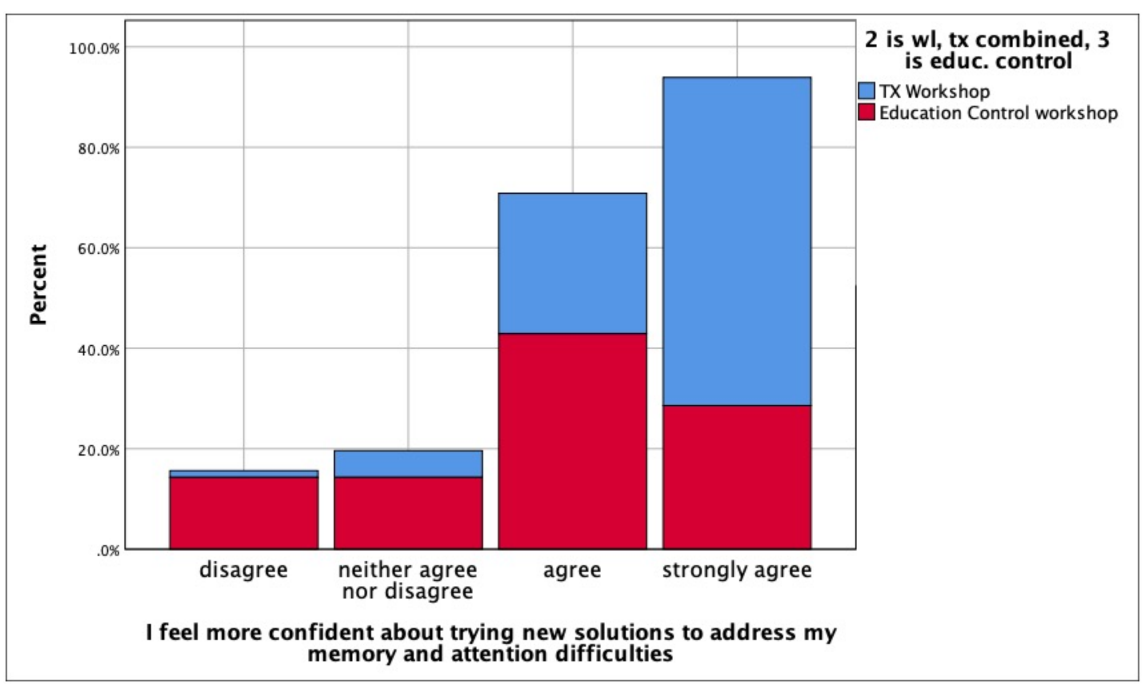


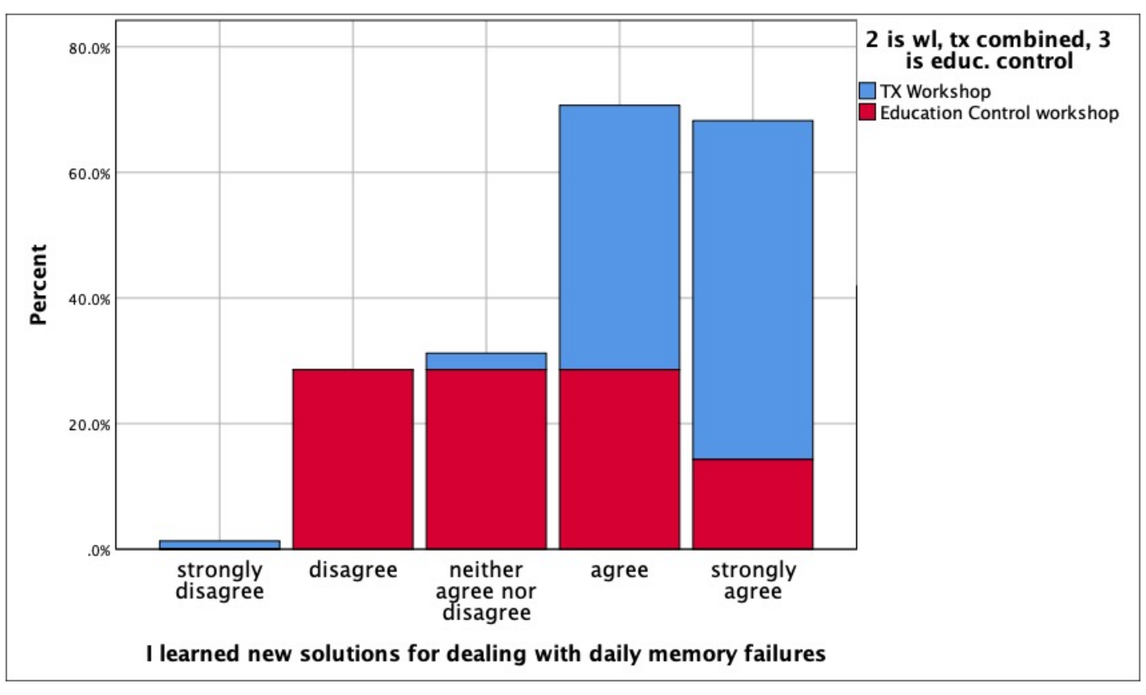


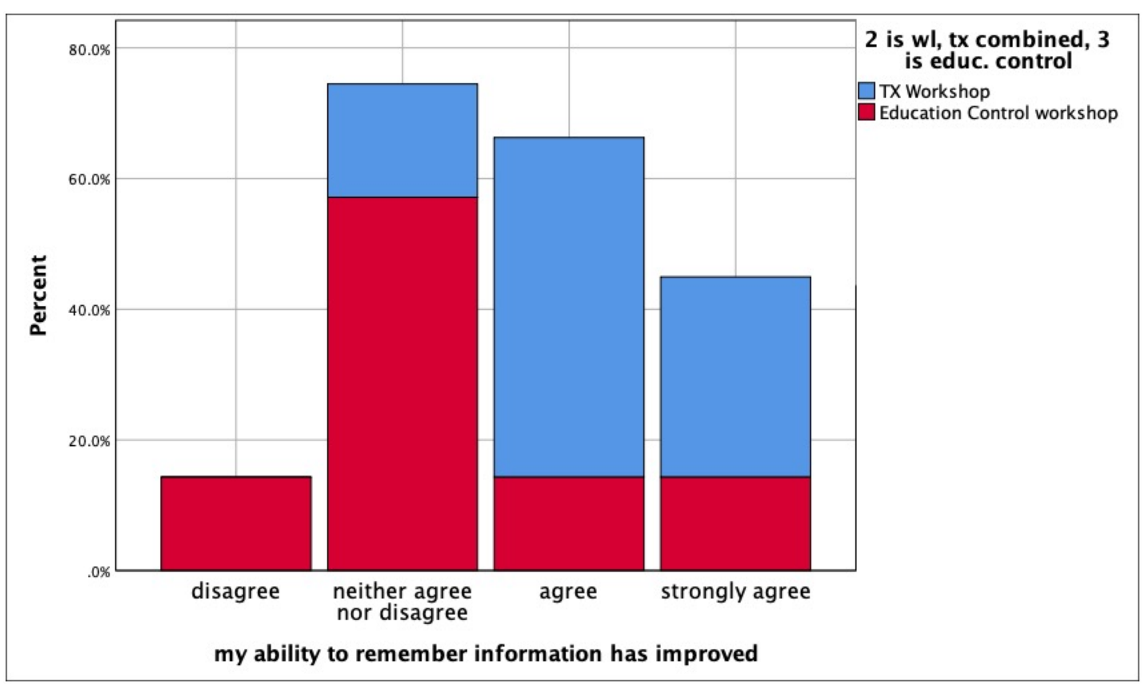


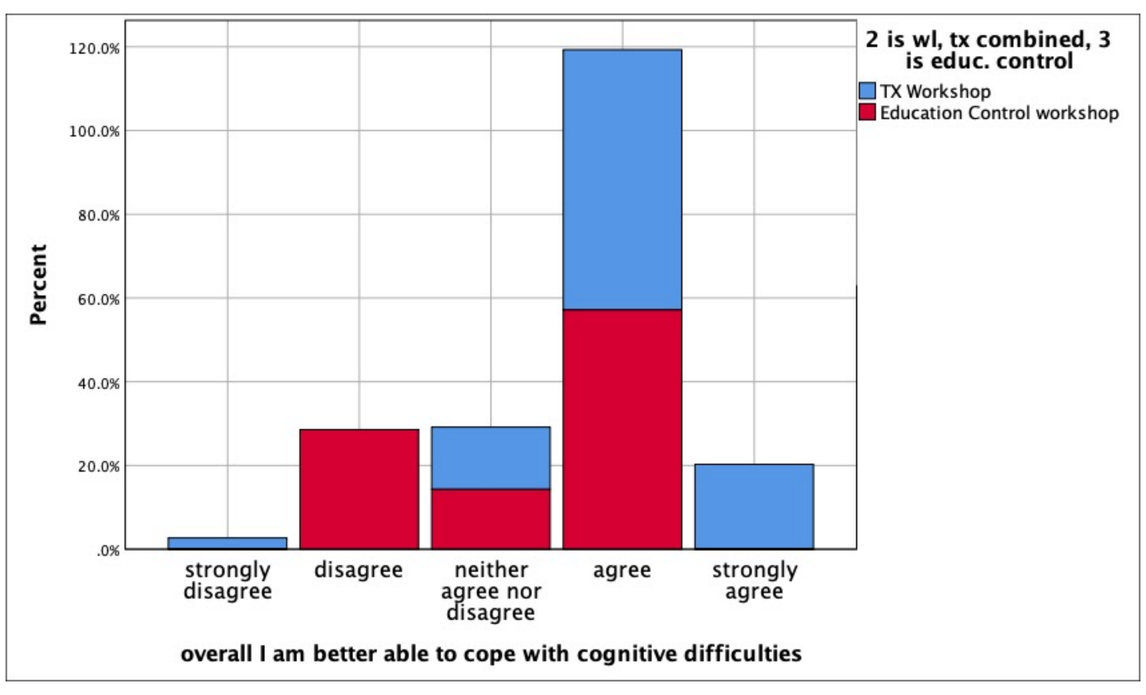


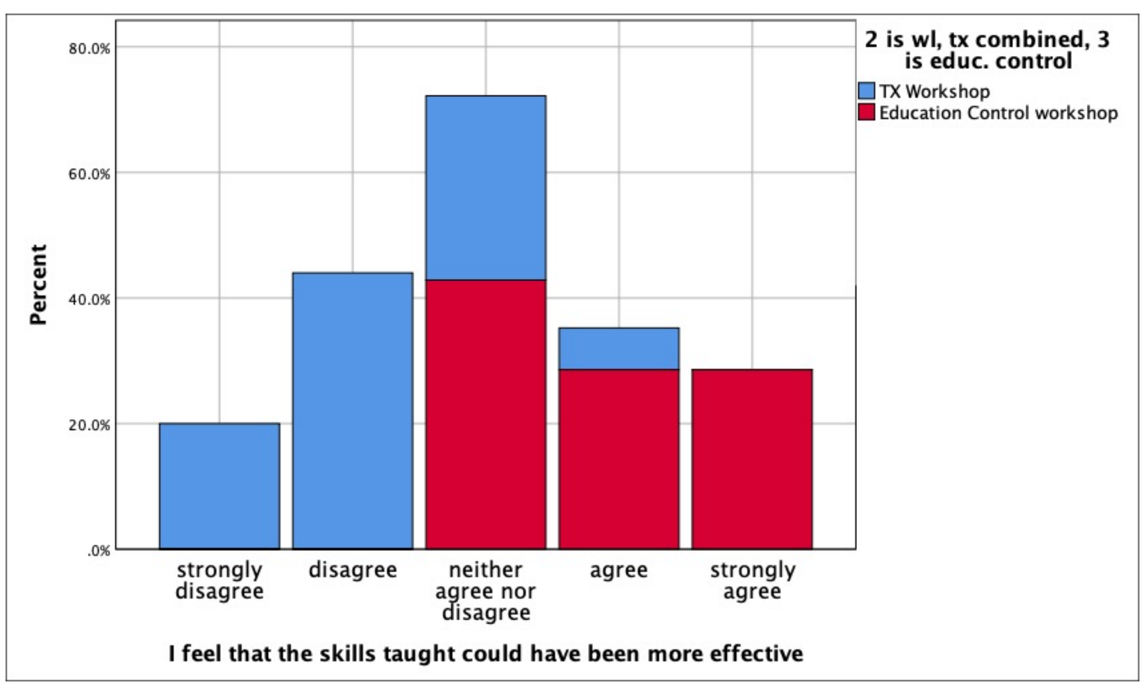


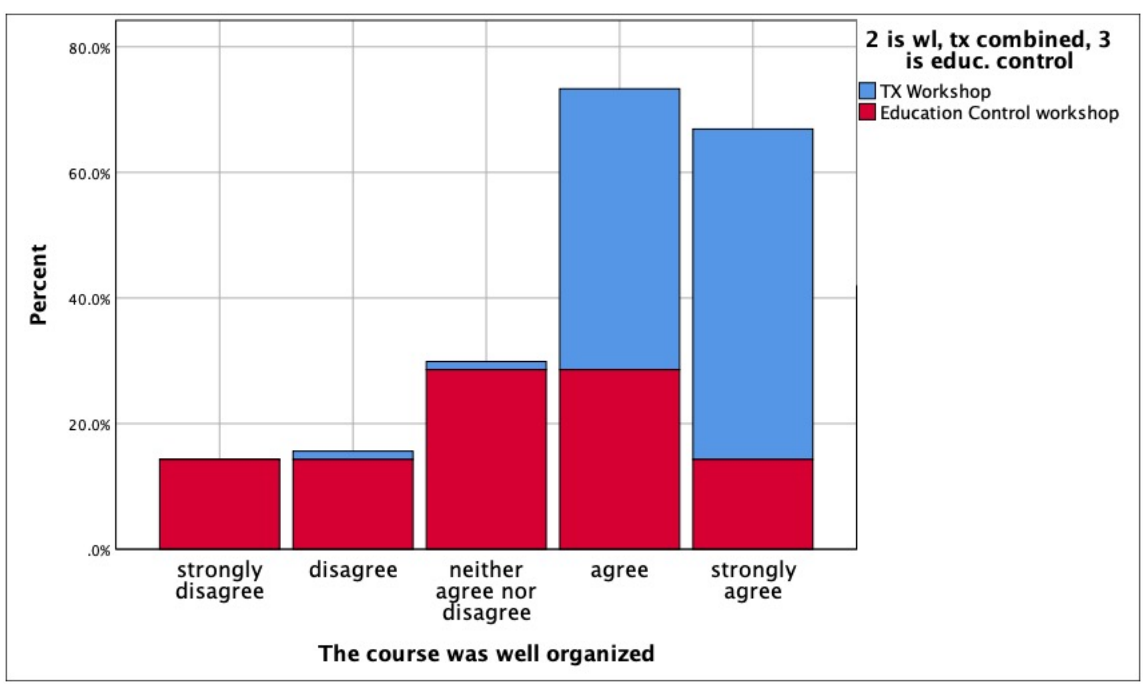


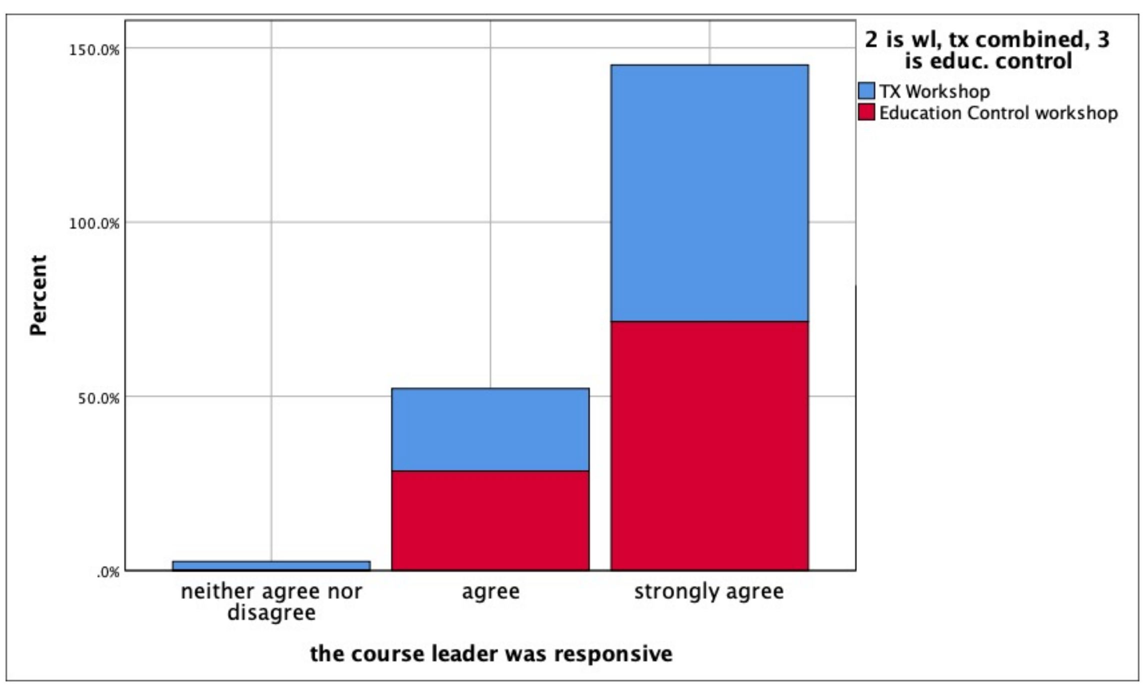


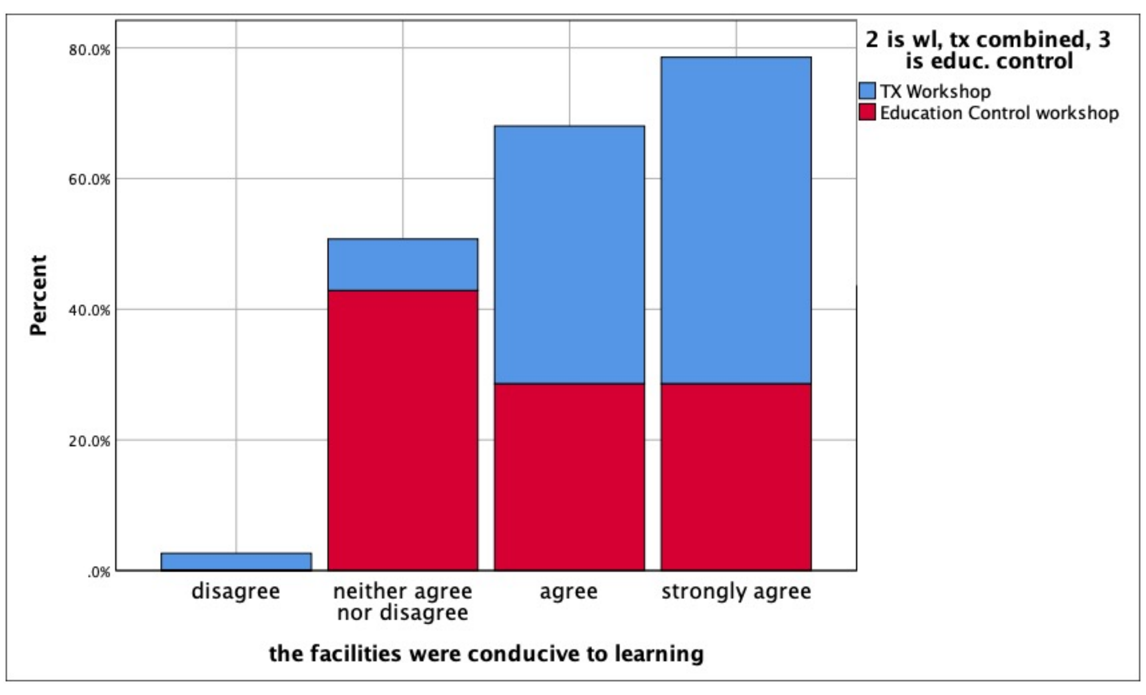


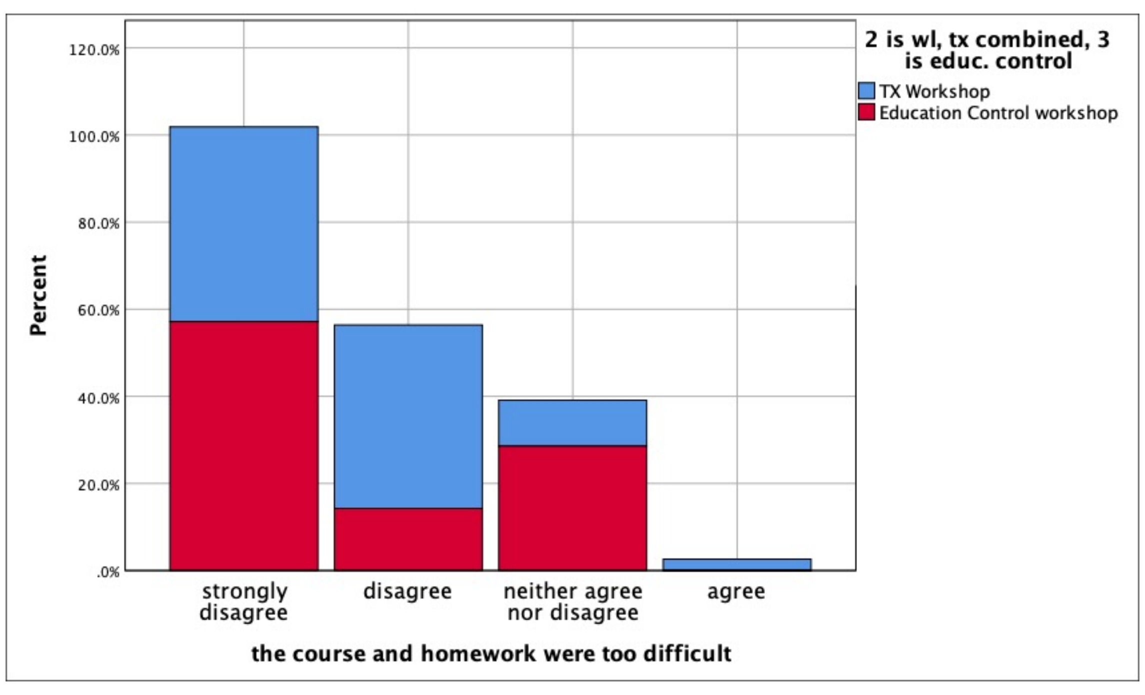


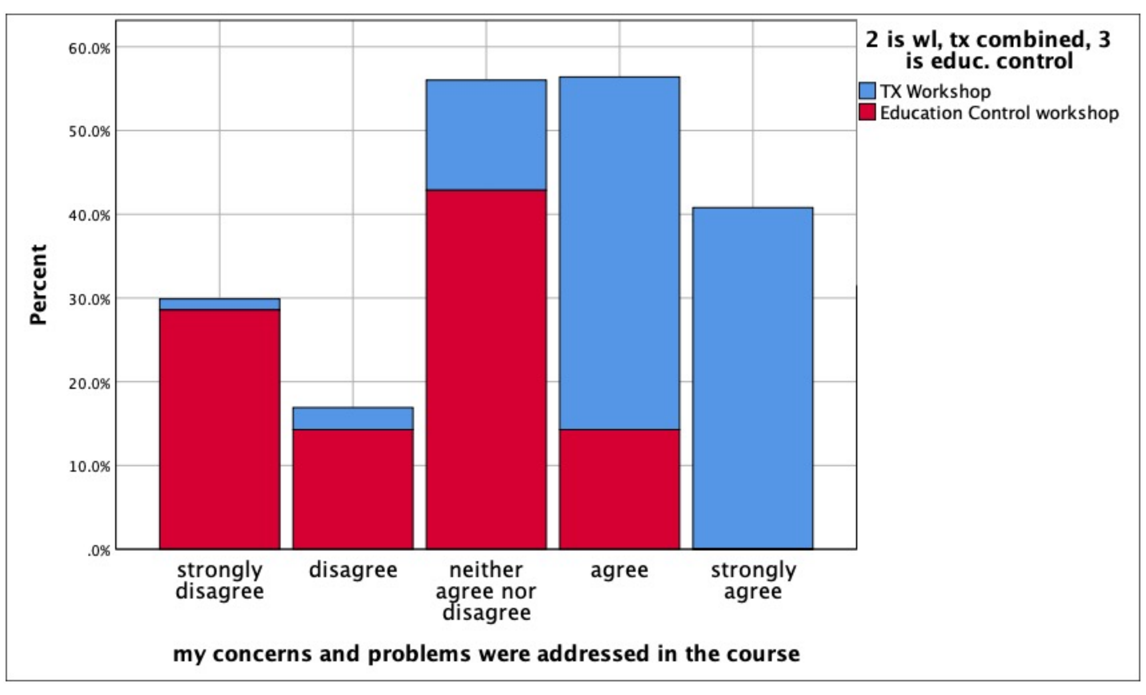


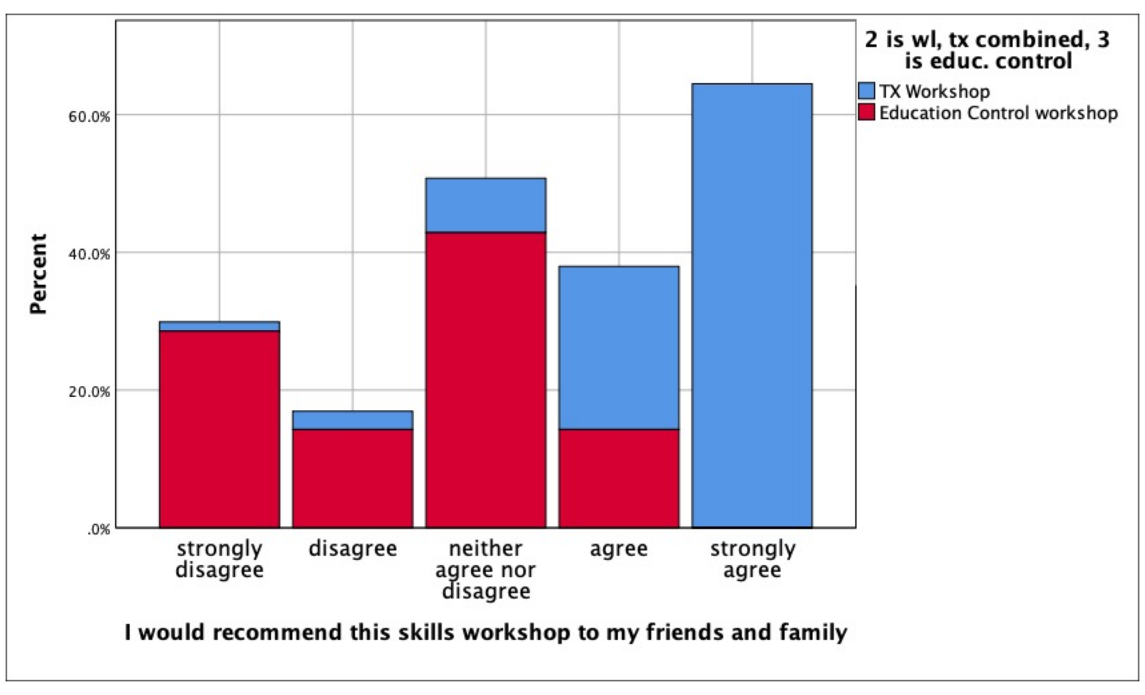


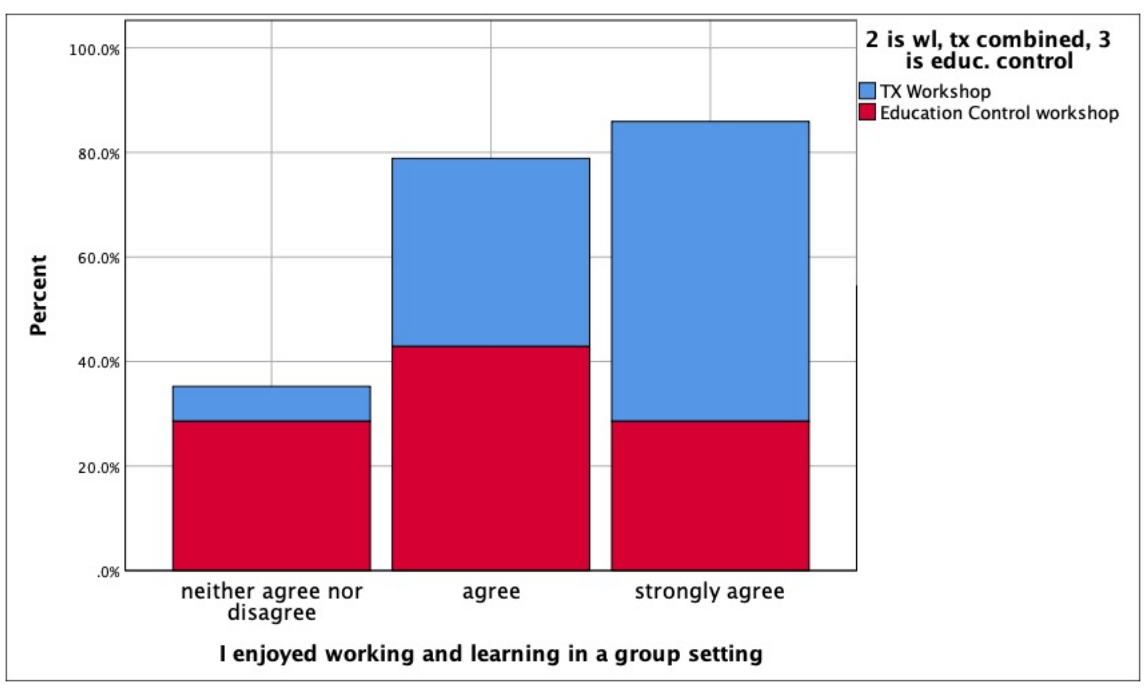


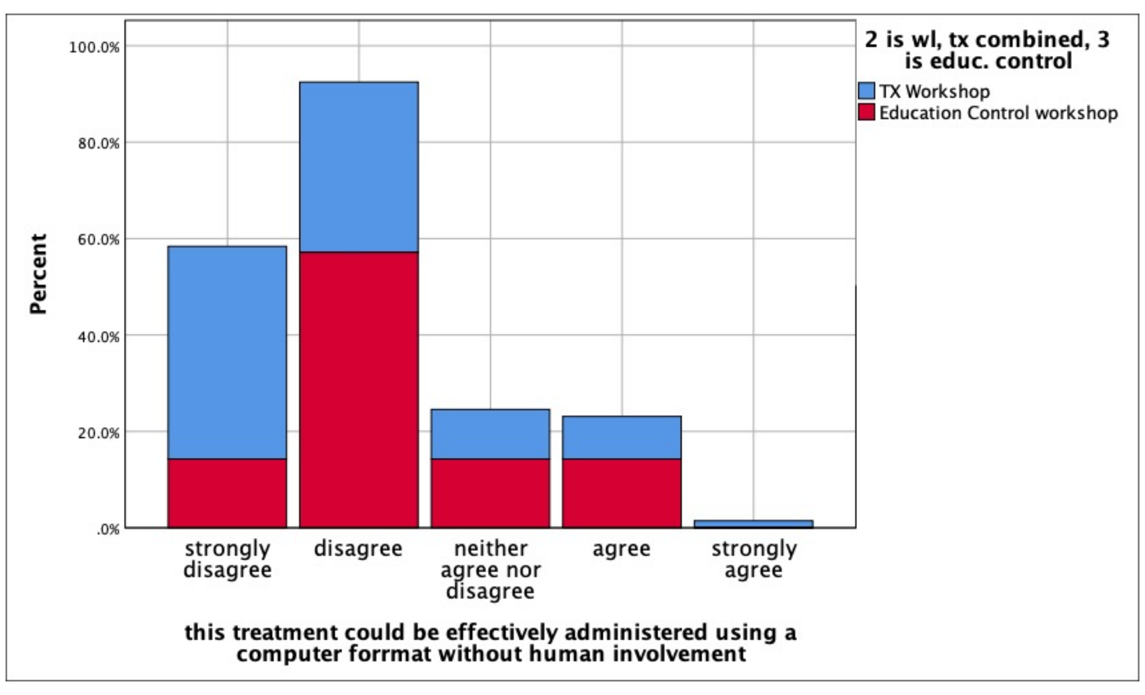


Figure legend: Figures are percent of total responses by group (TX vs EC) on the satisfaction questionnaire following workshop (TX or EC) completion. Blue bars (TX) sum to 100% and red bars (EC) sum to 100% . Missing bars indicate zero responses for that rating. TX group represents all participants who completed the TX workshops (i.e. a combination of participants with random assignment to TX as well as wait list (WL) participants who elected to participate in the TX workshop following completion of their WL condition. There is no statistical comparison for these results as there is no a-priori hypothesis
